# An alternative to petrochemicals: biomass electrovalorization

**DOI:** 10.1098/rsta.2023.0262

**Published:** 2024-09-23

**Authors:** Zamaan Mukadam, Soren B. Scott, Maria Magdalena Titirici, Ifan E. L. Stephens

**Affiliations:** ^1^ Department of Materials, Imperial College London, London, UK; ^2^ Department of Chemistry, University of Copenhagen, Copenhagen 2100, Denmark; ^3^ Department of Chemical Engineering, Imperial College London, London, UK

**Keywords:** electrochemistry, electrovalorization, biomass, platform chemicals

## Abstract

Replacing petrochemicals with refined waste biomass as a sustainable chemical source has become an attractive option to lower global carbon emissions. Popular methods of refining lignocellulosic waste biomass use thermochemical processes, which have significant environmental downsides. Using electrochemistry instead would overcome many of these downsides, directly driving chemical reactions with renewable electricity and revolutionizing the way many chemicals are produced today. This review mainly focuses on two furanic platform chemicals that are produced from the dehydration of cellulose, 5-hydroxymethylfurfural and furfural, which can be electrochemically reduced or oxidized to replace fuels and monomers that today are obtained from petrochemicals. Critical parameters such as electrode materials and electrolyte pH are discussed in relation to their influence on conversion efficiency and product distribution.

This article is part of the discussion meeting issue ‘Green carbon for the chemical industry of the future’.

## 1. Introduction

At present, many chemicals that are used in everyday life are sourced from petrochemicals. These include fuels, pharmaceuticals, adhesives and polymers [1,2]. The refinement of crude oil and the subsequent use of petrochemical products are associated with greenhouse gas emissions, which result in well-publicized detrimental impacts on the Earth’s climate [3]. The climate crisis has highlighted the urgent need to phase out fossil fuels if humanity is to avoid a climate catastrophe, by keeping global temperature rises below 2.0°C to prevent non-reversible negative effects on the environment [4]. The only certain way to achieve this is to stop extracting fossil fuels completely; however, this will require us to find an alternative source of chemicals.

Biomass can provide us with chemicals that can replace the ones we obtain from crude oil [5]. Carbon feedstocks obtained from biomass are more oxygenated than fossil fuel-sourced carbon, meaning the synthesis of oxygen-containing commodity chemicals requires fewer processing steps. About 170 billion metric tonnes of biomass is produced each year by photosynthesis, with about 75% of this consisting of carbohydrates, yet only 3–4% of this is used by humans for food and other uses [6]. Of course, not all of this biomass is readily and sustainably available, but this upper bound is clearly a vastly greater amount of carbon in comparison with the approximately 5.25 billion metric tonnes of crude oil extracted annually [7]. As a lower bound to available biomass, consider that global agricultural waste is estimated at 6.3 billion dry metric tonnes per year [8] and that global annual municipal waste, 60% of which is biomass [9], totals more than 2 billion metric tonnes per year [10]. It is estimated that around 7 billion metric tonnes of biomass are needed to satisfy bioenergy demands for future net zero scenarios based on the numbers estimated by the International Energy Agency [11]. The potential to eliminate reliance on petroleum by utilizing available biomass is clear.

**Figure 1.**
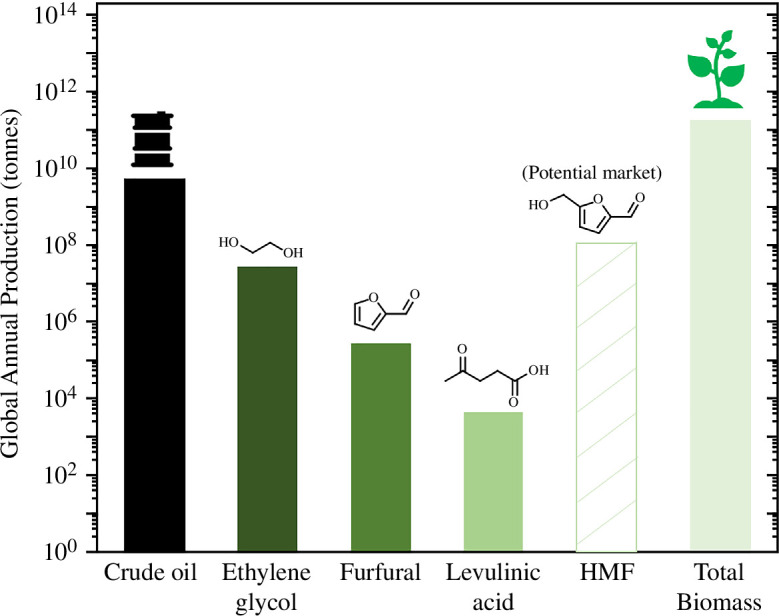
Annual production of biomass and crude oil feedstock, compared with annual production of the platform chemicals EG, furfural and LA. Data points were extracted from references [5–7,14–16].

Lignocellulosic biomass can be extracted from municipal biomass waste and is made up of a combination of hemicellulose (15–30%), cellulose (40–50%) and lignin (15–30%) [12]. Hemicellulose and cellulose are biopolymers consisting of five- and six-ring-membered carbohydrate monomers, which can be hydrolyzed into monosaccharides including glucose, xylose and mannose [13]. Further dehydrations produce useful furanic (C_5–6_ chemicals containing a furan ring) and straight-chain ‘platform chemicals’, which are defined here as chemicals that can undergo a variety of chemical reactions to produce even higher valued chemicals. Platform chemicals include furanic compounds including furfural and 5-hydroxymethylfurfural (HMF), as well as straight-chain platform chemicals such as levulinic acid (LA) and ethylene glycol (EG). The annual production of some of the platform chemicals is denoted in figure 1. In this figure, we depict an estimation of the potential annual production of HMF based on the annual production of terephthalic acid (TPA), due to one of the products of HMF oxidation being a direct replacement of TPA in polyethylene terephthalate (PET), which will be discussed later in this section. While this may not be completely accurate, it still highlights the scale of the untapped market of furanic platform chemicals in the near future.

In an ideal future scenario for a greener world, the plethora of chemicals that are currently sourced from crude oil refineries are instead derived from biomass, creating a ‘biorefinery’. This concept was developed during the late 1990s and is currently defined by the US Department of Energy as a processing plant where biomass feedstock is upgraded into a spectrum of valuable products [17]. The biorefinery encompasses many novel organic chemical reactions, processes, technologies and catalysts needed to synthesize chemicals that can replace petrochemicals.

Novel reactions in the biorefinery utilizing electrochemistry can be used to replace the thermochemical synthesis for biomass-derived platform chemicals, to lower the overall carbon footprint of chemical synthesis. Consequently, this can be called the ‘electrochemical biorefinery’. The resultant products of the reactions from the electrochemical biorefinery can be used to serve different purposes including fuels, solvents, polymers and others [18].

Platform chemicals with multiple oxidation/reduction products can have their selectivity and reaction efficiency governed by a combination of the electrode material, salt composition, electrolyte pH, applied potential and the initial concentration of the biomass feedstock. These parameters each have their own significance, which all need to be optimized for the most efficient synthesis [19]. Faradaic efficiency (ratio of the charge that is required to complete an electrochemical reaction compared with the total charge passed during the reaction) is naturally assumed to be the most important parameter to optimize as high efficiencies are desired. A reaction with high Faradaic efficiency towards a single product can aid in easing later separation steps, consequently lowering separation costs; however, a high current density is also preferred to make sure a reasonable production rate of desired products is achieved. The choice of solvent and identity of any counter ion is important and can influence the selectivity of products [20], as some platform chemicals are unstable and may degrade in extreme pH; however, the electrocatalyst material is usually the main parameter that affects product selectivity. Electrode materials for these reactions can consist of nanoparticles of different loadings. For a future scale-up of electrosynthesis, the cost of precious metals must also be considered; however, the cost of the entire process is also important. For example, in proton electrolyzer membranes, the cost of the scarce iridium electrodes is only around 3% of the entire system [21], suggesting that the cost of the catalyst may not necessarily be the most important factor to optimize. Furthermore, most reactions reported in the literature describe biomass electrovalorization on a small benchtop scale. For the eventual industrial scale-up of these processes, a flow reactor would be preferable for a continuous synthesis, such as ones reported for electrochemical amino acid production [22] and electrochemical CO_2_ reduction [23]. In addition, methods to separate products need to be considered to lower production costs. In CO_2_ electrolyzers, Jouny *et al.* [24] determined that selectivity and overpotential were the most significant factors to optimize for high-electron products, whereas current density was the least significant. These results highlight that the optimization of parameters is dependent on the targeted product. In addition, product separation, storage, transportation and electricity costs also need to be considered [25]. Advances in these parameters in recent years point towards these challenges eventually being overcome as technology improves, and also as the cost of renewable electricity becomes cheaper [26].

Furfural is a C_5_ furanic aldehyde produced by the dehydration of hemicellulose [5]. The reduction of furfural can yield high-valued chemicals such as furfuryl alcohol (FAL) and 2-methylfuran (2-MF), which are used in polymers and biofuels [27]. Furthermore, furfural can be oxidized into furoic acid, which has uses in renewable polymers too [28].

**Figure 2.**
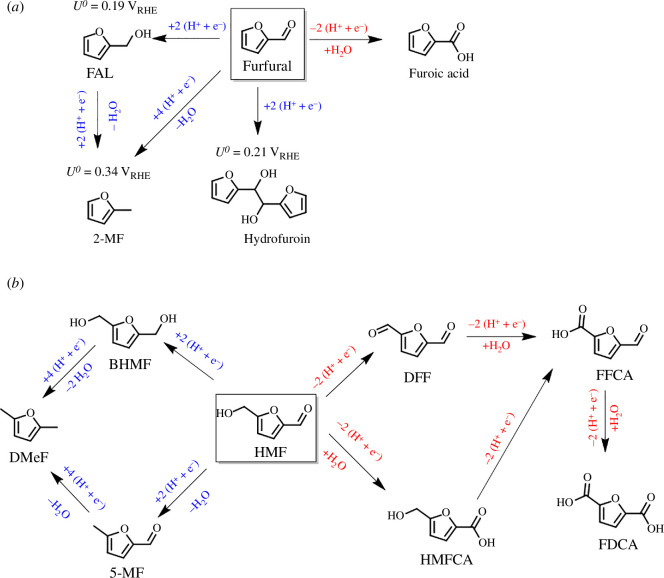
Possible oxidation and reduction products available from (*a*) furfural and (*b*) HMF. Red labels depict electrochemical oxidation products, and blue labels depict electrochemical hydrogenation products. BHMF (2,5-bishydroxymethylfuran), 5-MF (5-methylfurfural), DFF (2,5-diformylfuran), HMFCA (5-hydroxymethyl−2-furancarboxylic acid), FFCA (5-formyl−2-furoic acid). Δ_*f*_*G*^0^ values for calculating electrode potentials (*U*^0^) obtained from references [29,30].

HMF is a C_6_ furanic compound (figure 2) produced from the dehydration of cellulose [31]. HMF can be oxidized into 2,5-furandicarboxylic acid (FDCA), which has attracted attention as it can be used as a replacement monomer for TPA in PET to produce polyethylene furanoate polymers. FDCA was identified by the US Department of Energy as one of the most important chemicals for a sustainable future [32]. About 30.47 million metric tonnes of PET was produced in 2019 [33] and is mostly used in the beverage packing industry. The TPA used to make PET is currently obtained from the oxidation of *p*-xylene, which is sourced from non-renewable petrochemicals. Aside from uses in polymers, the reduction products of HMF can yield 2,5-dimethylfuran, which has a high energy density and can be used as a biofuel [34]. The vast number of compounds available from HMF makes this platform chemical extremely attractive for the future.

## 2. Electrochemical reduction reactions

The reduction of furfural produces useful chemicals such as FAL, 2-MF and hydrofuroin, as well as their fully hydrogenated derivatives if the furan ring itself is reduced. However, the extra thermodynamic stability of the furan ring makes the complete reduction of these compounds unfavourable under ambient conditions [35] and has only been reported in small amounts (8% Faradaic efficiency for 2-methyltetrahydrofuran) on Pd electrodes at high overpotentials [36]. Hence, for this review, the focus is on the reduction of the substituted functional groups. Furfural reduction reactions are given more focus here due to the greater usefulness of their products.

### (a) Factors governing furfural reduction selectivity

#### (i) Electrode material

**Figure 3.**
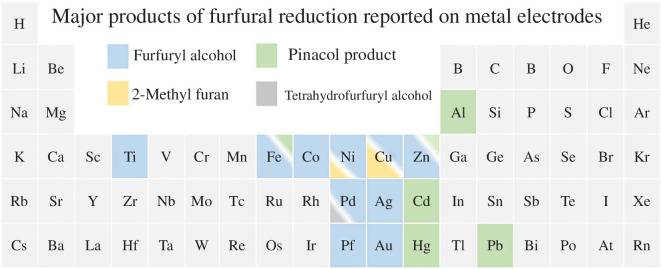
Summary of major reported furfural electrochemical reduction products on monometallic electrodes at moderate cathodic potentials. The ratio of the shaded squares depicts the major and minor products. The pinacol product represents hydrofuroin. Cu electrodes are the only monometallic species that produce 2-MF with high Faradaic efficiencies [37–45].

**Figure 4.**
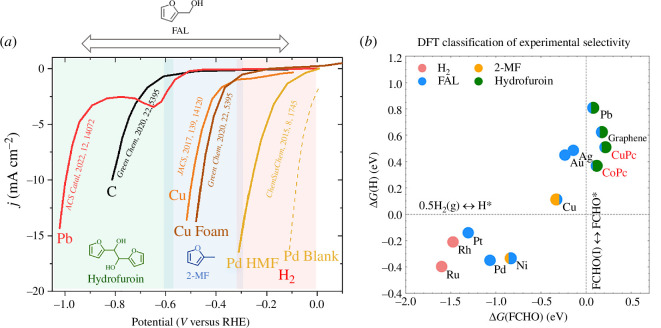
Electroreduction products of furanic compounds on different electrodes. (*a*) Co-plotted linear sweep voltammograms of different metal cathodes for the electrochemical reduction of furfural or HMF in acidic electrolytes. Shaded areas depict a major reduction in productions reported [48–51]. (*b*) Adsorption energies of furfural and hydrogen obtained using density functional theory (DFT) and reported products. Co/CuPc depict metal phthalocyanines [30].

Figure 3 [46] shows a comparison of the electrochemical selectivity of various metal electrodes for furfural reduction. While the number of electrodes that can produce FAL is more diverse, the production of hydrofuroin is limited to Al, Hg, Pb and Cd electrodes, and for 2-MF only, Cu electrodes have been reported to produce 2-MF with selectivities above 80% Faradaic efficiency. While Nilges & Schroeder [39] have reported up to 28% Faradaic efficiency for 2-MF production on Ni electrodes, Cu electrodes are the present state-of-the-art for 2-MF production and report Faradaic efficiencies of over 80% and high current densities at relatively low operating potentials of −0.50 to −0.75 V versus the reversible hydrogen electrode (RHE) [39]. This is advantageous as Cu is a cheap transition metal and is relatively abundant. For electrochemical hydrofuroin production, the best-reported electrodes are carbon and Pb, which have shown between 60 and 70% Faradaic efficiency at mild cathodic potentials (figure 3) [39]. The effect of the electrode material on selectivity may be rationalized by relative binding affinities between an organic substrate, the electrode surface and hydrogen [47]. In brief, we can come to an initial observation that if a surface binds hydrogen too strongly, the hydrogen evolution reaction (HER) will likely dominate, whereas weaker hydrogen binding may prevent surface-level hydrogenation altogether leading to radical coupling, and a balance between the two can result in more effective electrochemical reductions, i.e. for 2-MF selectivity. This is roughly outlined in figure 4*a,b*, which depicts the theoretical binding energies of furfural with hydrogen and the linear sweep voltammograms of reported electrodes, outlining the reduction products seen on each. It is important to reacknowledge that selectivity can be governed by a combination of parameters, for example, Pb electrodes produce 2-MF at very cathodic overpotentials in acidic media [52].

#### (ii) Effect of pH

**Figure 5.**
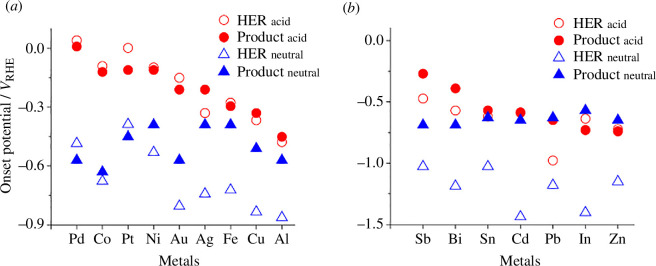
Comparison of the onset potentials of (*a*) transition metals and (*b*) post-transition metals with regard to the HER and HMF reduction, in acidic (0.5 M H_2_SO_4_) and neutral (pH 7 phosphate buffer) media. The ‘product’ on the legend describes HMF reduction products. Onset potentials of the HER and HMF reduction were reported by recording the potential at which a current density of −0.5 mA cm^−2^ was achieved [55].

The pH of the electrolyte is an important parameter that can be tuned to target specific reduction products. For 2-MF production, an acidic electrolyte is a necessity on Cu, which was proven by Liu *et al*. [53] by conducting furfural reduction in pH 10 and only seeing FAL as the main product with over 70% Faradaic efficiency. At low pH, the competing HER is more active compared with alkaline electrolytes [54]. An explanation for this was suggested whereby the activation barrier for HER is lower at this pH [49,54]. At a higher pH, there is a lower concentration of H^+^ and in turn a lower chance of inducing unwanted HER steps. From this, it is possible to hypothesize that running the electrochemical reduction reactions at basic pH could be one possible option to suppress the HER and increase overall efficiency towards organic reduction products. However, furanic compounds are known to degrade at high pH, which will be discussed in greater detail further into this review. In figure 5, it is seen that HMF hydrogenation and HER tend to have similar onset potentials (defined here as the potential needed to achieve a certain current density) at low pH [55]. There is a greater difference in the onset potentials between HER and HMF reduction at neutral/basic pH, but in acidic pH, the onset potentials of the HER and of HMF electroreduction are less cathodic than when compared with neutral media.

The effect of acidic pH on 2-MF production was seen on Cu electrodes by Jung & Biddinger [20], whereby electrochemical furfural reduction was tested in 0.1 M NH_4_Cl (pH 3.4), 0.1 M H_2_SO_4_ (pH 1.1) and 0.5 M H_2_SO_4_ (pH 0.5), at a constant current density of −10 mA cm^−2^. 2-MF had the highest Faradaic efficiency at pH 0.5 (31%) and decreased as the pH increased, with less than 2% Faradaic efficiency of 2-MF being recorded at pH 3.4 [20]. The authors also saw this effect using different concentrations of HCl, which suggested that this effect was due to pH and not the nature of the anion [20]. By contrast, the selectivity for FAL increased significantly when the pH was raised to between pH 6 and 10. Brosnahan *et al.* [43] also observed the same phenomenon while studying furfural reduction using Ag_60_Pd_40_ nanoparticles. To selectively produce FAL, a neutral pH was preferred due to lower onset potentials for furfural reduction, and low amounts of HER in these conditions compared with other electrolytes, namely the acidic electrolytes that showed increased levels of HER. Interestingly, neutral K^+^ and Na^+^ phosphate buffers showed no difference in FAL selectivity, which contrasts the electrochemical CO_2_ reduction reaction, in which product distribution is directly affected by the choice of cation [56].

#### (iii) Other effects on furfural reduction selectivity

**Figure 6.**
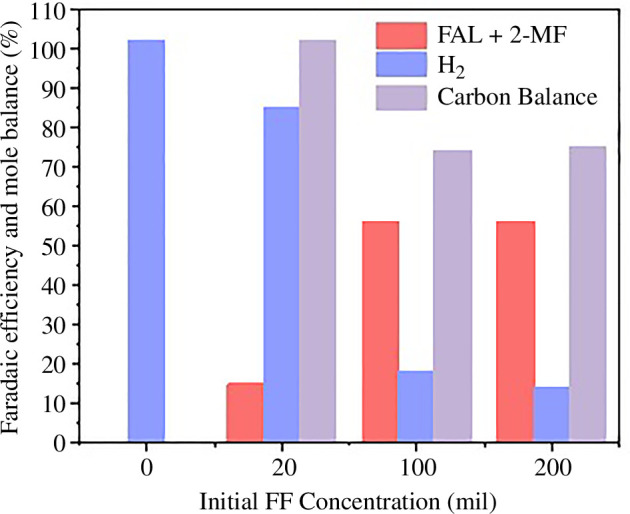
Products from electrochemical furfural reduction with changing initial furfural concentration. Reaction conditions: 0.5 M H_2_SO_4_ electrolyte with 4:1 ratio of acid to acetonitrile co-solvent, Cu foil electrode, 1.5 h reaction time at −0.80 *V*_RHE_. Figure adapted from reference [57].

The initial concentration of furfural is another factor that was shown to play a role in controlling the selectivity of furfural reduction products by Jung *et al*. [57] in acidic media (0.5 M H_2_SO_4_ with acetonitrile co-solvent). At low initial furfural concentrations, H_2_ gas was the main product, and the combined FAL and 2-MF Faradaic efficiency increases with increasing starting concentrations of furfural, alongside decreasing HER rates (figure 6). The addition of acetonitrile as a co-solvent is useful as it helps solubilize organic compounds with its high dielectric constant (37.5). Ledezma-Yanez *et al*. [58] showed that acetonitrile may adsorb on an electrode surface and affect HER rates. In electrosynthesis, the addition of acetonitrile can lower the formation of humins as water is critical for ring-opening reactions [59]. Parpot *et al*. observed humins formed as a by-product in high concentrations of furfural 0.1 M KOH (pH 13) on Cu electrodes [60]. It is seen that by increasing the initial concentration from 0.25 to 0.50 M, the total yield of furanic products decreased by 27%.

**Figure 7.**
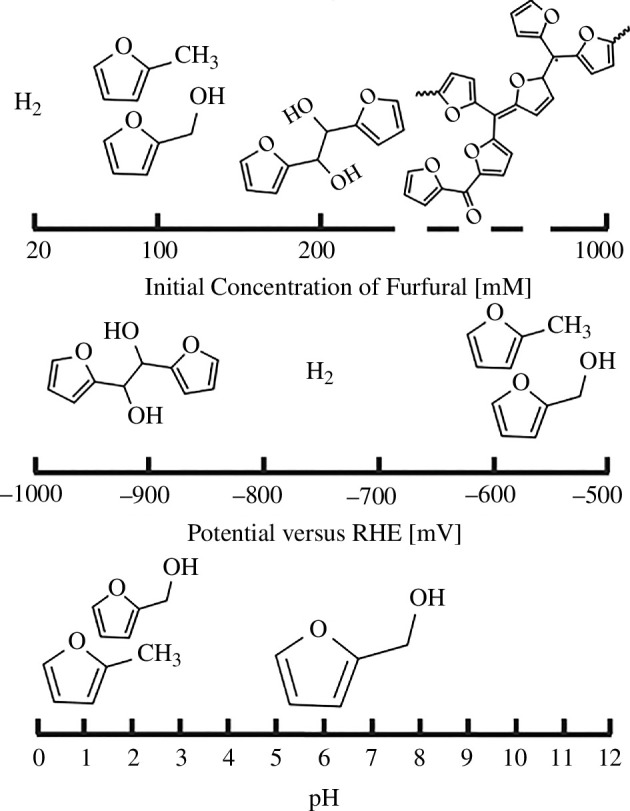
Qualitative selectivity predictions of furfural reduction products on Cu electrodes. The size of the molecules represents the relative abundance expected for each parameter [19].

Raman studies were used by Li & Kornienko [61] to study furfural reduction on different electropolished Cu single crystals (111, 110, 100 and roughened Cu), at −0.526 *V*_RHE_ in acidic electrolytes. The study concluded that the Cu(110) surface produced FAL with the highest Faradaic efficiency (up to 80%), whereas the roughened Cu exhibited 2-MF as the major product. The authors suggested that this result is due to the increased number of defects in the roughened surface providing binding sites for a CH_2_OH group to adsorb, straining the bond and making it easier to cleave off. A qualitative representation for furfural reduction on Cu was produced by May & Biddinger [19] (figure 7) depicting the expected products that should be produced under certain parameters. Although the qualitative representations are from experimental results in their group, we could conjecture that some general trends can be used to explain or possibly predict results on other electrodes. For example, the humin products at very high initial furfural concentrations may be independent of electrode materials, due to the general instability of furfural, especially in alkaline pH.

### (b) Mechanisms of carbonyl bond electroreductions

Understanding the mechanisms behind electrochemical reduction reactions can provide us with invaluable information to help produce more selective electrochemical syntheses in the future. For furfural reduction, these can be separated into two categories: carbonyl bond reduction to form FAL and 2-MF, and radical coupling to produce hydrofuroin.

#### (i) Carbonyl bond reduction

**Figure 8.**
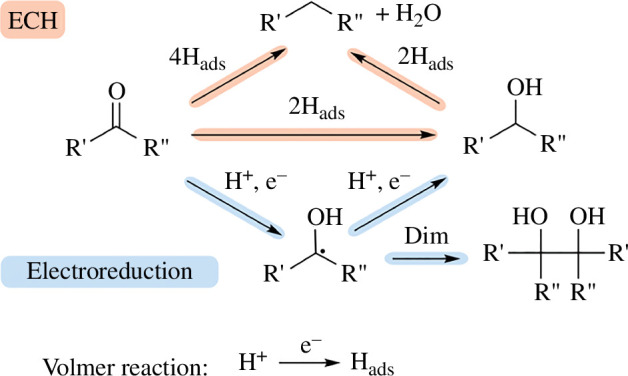
Proposed electrochemical reduction pathways of carbonyl compounds. The red pathway depicts the ECH pathway using adsorbed hydrogens, whereas the blue pathway depicts the PCET pathway [51].

Following the adsorption of furfural, two possible mechanisms for reduction are possible, which include a direct reduction of the carbonyl bond using adsorbed hydrogens (ECH mechanism) or a proton-coupled electron transfer (PCET) using H^+^ ions in solution (electroreduction mechanism). An electrochemical hydrodeoxygenation is also possible and can remove oxygen atoms on furfural as water. Work from Chadderdon *et al*. [51] suggested two possible pathways of electrochemical carbonyl reduction in aqueous media on Cu electrodes (figure 8). The authors suggested an outer-sphere pathway where parts of the reduction do not occur on the electrode surface in the case of direct electroreduction (blue pathway; figure 8) and an inner-sphere pathway whereby the reduction of the carbonyl group is achieved completely on the electrode surface using adsorbed hydrogens (red pathway; figure 8).

#### (ii) Reductive radical coupling

The proposed outer-sphere electroreduction mechanism (blue pathway; figure 8) shows the potential for producing radical species that can self-dimerize to produce a pinacol product. This is a possible method of producing C–C bonds under mild conditions in contrast to popular methods of thermochemical C–C couplings done via expensive Pd-catalysed Suzuki [62] or Stille reactions [63]. By coupling two furfural molecules together, hydrofuroin can be produced, which is attracting attention as a potential drop-in jet fuel [48].

Electrochemical furfural reduction on Pb, Al and carbon electrodes was studied by Nilges & Schroeder [39]; they reported greater selectivity towards hydrofuroin than Cu. Chadderdon *et al*. [51] also reported results for furfural reduction on Pb electrodes in acidic electrolytes with about 30% Faradaic efficiency for hydrofuroin, but there were many unidentifiable products that were attributed to unselective radical coupling to form oligomers that can poison the electrode. Following this, it could be further suggested that the furfuryl radicals are coupled on the electrode surface without the use of any adsorbed hydrogen, as Pb has a notoriously large overpotential for HER due to H binding very weakly on this surface [64]. This was in contrast to a study by Lie *et al*. [49], whereby a combination of electron paramagnetic resonance spectroscopy and theoretical techniques identified the furfuryl radical in the electrolyte solution on Pb electrodes in acidic pH. This conclusion suggests that there may be a secondary mechanism for electrochemical reductive coupling reactions; however, further detailed literature on solution coupling mechanisms was limited. Furthermore, outer-sphere reactions were explored by Chadderdon *et al*. [51] using self-assembled monolayers to cover the binding surface of copper electrodes for furfural reduction aiming to facilitate only outer-sphere reactions. This resulted in zero 2-MF being produced and minor amounts of FAL and hydrogen as the major products.

**Figure 9.**
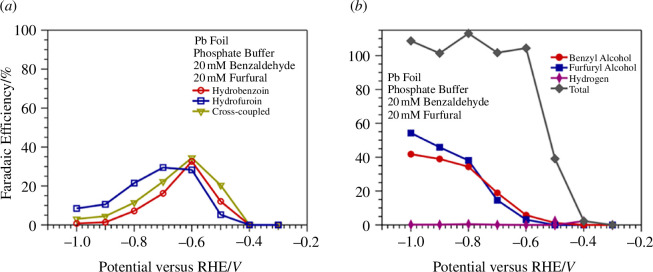
Electrochemical reduction of furfural and benzaldehyde on Pb foil. Faradaic efficiencies of (*a*) coupled products and (*b*) reduced alcohol products after electrolysis. Reaction conditions: 0.25 M sodium phosphate buffer (pH 6.7), 20 mM of hydrofuroin and benzaldehyde [66].

Conductive carbon materials as electrodes are attractive options for electrodes because carbon is cheap and ubiquitous in nature. The dimerization of HMF was seen on carbon electrodes by Kloth *et al*. [65] in alkaline electrolyte, which produced the coupled species 5,5'-bis(hydroxymethyl)hydrofuroin with Faradaic efficiencies between 32 and 35%, but the other 65–68% of consumed HMF were unidentifiable products. This result was also seen in similar experiments of HMF electroreduction on carbon electrodes by Chadderdon *et al*. [43] where over 50% of the consumed HMF was also unidentifiable. Both authors suggested it was likely that unselective radical coupling occurred, which produced oligomers after the single electron transfer to produce the radical in the first step. Other carbon-containing materials were also reported in this study including an experiment using Ag/C cathodes [43]. While the main product detected was the diol 2,5-bishydroxymethylfuran (BHMF), some experiments reported that BHMF made up only about 50% of the total product distribution, with the rest being unidentifiable. While it could be argued the unidentifiable humins are from the Ag, it cannot be ruled out that secondary mechanisms occur on the carbon material that produces BHMF and oligomer products. The scope of electrochemical radical coupling was extended further by Anibal & Xu [66] who attempted the heterocoupling of two aldehydes (furfural and benzaldehyde) on Pb electrodes in a near-neutral electrolyte. Maximum Faradaic efficiency of 38% at −0.60 *V*_RHE_ of the heterocoupled product was observed, with the rest of the products being the homocoupled hydrofuroin or hydrobenzoin. The effect of applied potential on selectivity was seen when, at more cathodic potentials, the rate of radical coupling decreased and the aldehyde reduction into alcohols was the dominant reaction (figure 9). Electrochemical cross-coupling between furfural and LA was also demonstrated by Wu *et al*. [67] but at an anodic potential of 1.41 *V*_RHE_, using Ni_3_N particles in alkaline media. A selectivity of 80% of the coupled product was recorded at this potential.

Selective electrochemical HMF reduction can produce BHMF and was reported on AgCu foam cathodes with up to 82% Faradaic efficiency by de Luna *et al*. [68]. Analogous to furfural electroreduction, homocoupling of HMF was demonstrated and was also seen on Ag/C electrodes in a separate study by Dai *et al*. [69]. The auxiliary reaction at the anode for most reported biomass electroreduction reactions is water oxidation, which has a relatively high thermodynamic potential (1.23 *V*_RHE_). The thermodynamic potential required for biomass oxidation reactions is typically less positive than 1.23 *V*_RHE_, which could, in principle, lower the overall energy demand of the system. Conversely, while hydrogen production at the cathode yields a valuable chemical, the production rate of hydrogen from these systems compared with commercial water electrolyzers is far lower due to the limitations in current densities at the anode. Hence, producing useful commodity chemicals such as BHMF paired with electrochemical biomass oxidation reactions may be a more attractive option.

## 3. Electrochemical oxidation

**Scheme 1.**
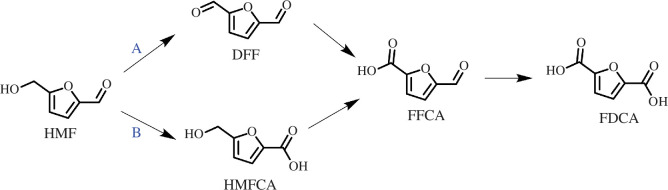
Competing pathways for HMF oxidation to FDCA.

A new library of products is available from the electrochemical oxidation of HMF using anodic potentials, which can produce the HMF oxidation products: DFF, HMFCA, FFCA and FDCA (scheme 1). Furoic acid, a result of furfural oxidation, has uses in renewable polymers and has been reported on Pt [70] and Au [71] electrodes in acidic media, with minor amounts of maleic acid produced via ring-opening reactions. While Faradaic efficiencies reached over 80%, total current densities were low, at around 1–10 µm cm^−2^. Higher current densities for furfural oxidation on the mA cm^−2^ scale were seen using CuNi nanoparticles in 1 M KOH [72]. While high current densities were observed, a low selectivity was seen. In this context, furfural oxidation was used as a paired reaction with the HER, resulting in a lowered energy demand for green hydrogen production compared with other auxiliary reactions such as water oxidation.

### (a) Significance of pH

There are fewer reports of electrochemical oxidation of furfural in the literature, likely due to the greater usefulness and prices of HMF oxidation products compared with furfural oxidation products. For this reason, this section will mainly focus on the electrochemical oxidation of HMF. Like electrochemical reduction reactions, the electrode material and the pH of the electrolyte can affect the selectivity and activity of oxidation reactions [73]. For electrochemical alcohol oxidation, the preference for alkaline electrolytes was highlighted by Kwon *et al*. [74] who reported that the first step of the reaction is an outer-sphere base-catalysed deprotonation to produce a reactive alkoxide species. In addition, it is observed that the maximum oxidation activity for a range of alcohols was achieved at a pH equal to the pK_a_ of each alcohol. In this regard, for ethanol oxidation on Au electrodes, the oxidation activity was orders of magnitude higher in pH 13 than that of acidic pH, supporting this notion [74].

### (b) Electrode material

**Figure 10.**
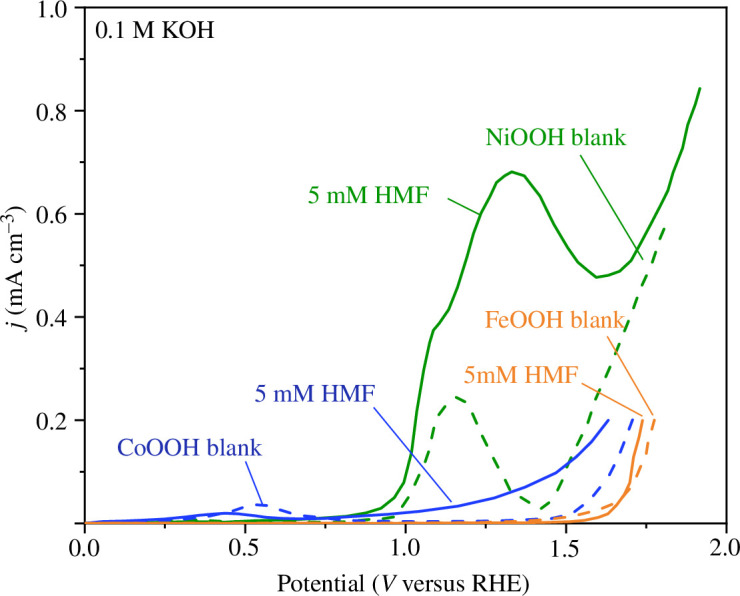
LSVs of NiOOH, CoOOH and FeOOH before (dashed line) and after (solid line) the addition of 5 mM HMF. Reaction condition: 0.1 M KOH (pH 13), at room temperature, scan rate was 5 mV s^−1^. Data points were taken from reference [76].

The disubstituted nature of HMF results in two competing oxidation pathways (scheme 1), which adds an extra layer of complexity in controlling selectivity. The choice of electrode materials can be a major factor in determining which route HMF oxidation products take. HMF electrooxidation was reported as early as 1991 by Grabowski *et al*. [75] using Ni/NiOOH foam electrodes in 1 M NaOH electrolyte (pH 14), reporting a 71% product yield for FDCA production, with current densities as high as 16 mA cm^−2^ and 84% Faradaic efficiency. Other anode materials such as oxyhydroxides consisting of NiOOH, CoOOH and FeOOH were also reported for HMF oxidation activity by Taitt *et al*. [76] where it was seen that NiOOH films were the most efficient catalyst with reported FDCA selectivity and Faradaic efficiency at 96% in a 0.1 M KOH electrolyte (pH 13). The LSVs of each catalyst are denoted in figure 10 and show the superior performance of NiOOH compared with the others [76]. Latsuzbaia *et al*. [77] tested NiOOH foams for a continuous, industrial scale-up of HMF oxidation and reported high selectivity (90%) and Faradaic efficiency (80%) for FDCA at pH 12.

**Figure 11.**
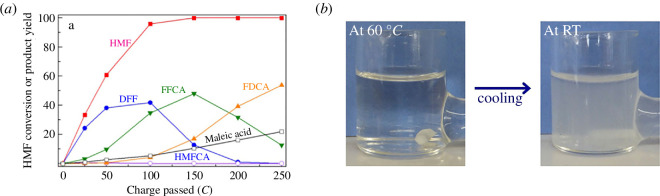
The electrochemical oxidation of HMF in acidic electrolyte at 1.60 *V*_RHE_ using MnO_x_ electrodes. (*a*) Products obtained from HMF oxidation at different amounts of charge passed. (*b*) Precipitation of FDCA in solution after cooling from 60°C. Figure reprinted with permission from the publisher [79].

Vuyyuru *et al*. [78] attempted to oxidize HMF on PtO_x_ electrodes, which produced DFF at 18% Faradaic efficiency as the main organic product. PtO_x_ electrodes exhibited some HMF oxidation activity at low pH whereby it was reported a 4% yield of DFF was obtained at 2.0 *V*_RHE_ in a pH 1.06 electrolyte [77]. The remaining approximately 96% of HMF consumed was unaccounted for and was labelled as HMF degradation products. This result was also seen by Kubota *et al*. [79], whereby DFF was produced at just 8% Faradaic efficiency on PtO_x_ electrodes at acidic pH, with large amounts of HMF being consumed yet unaccounted for during quantification. One compound that was detected from this HMF degradation was maleic acid, which is a precursor to succinic acid, a dicarboxylic acid that can be used in renewable polymers [80]. The same study also used a MnO_x_ anode at 60°C, to achieve FDCA at an impressive Faradaic efficiency of 53.8% at 1.60 *V*_RHE_ [79]. The synthesized FDCA spontaneously precipitated in the electrolyte due to the insolubility of FDCA in acidic media. This spontaneous precipitation of FDCA is useful because it negates the need for a reagent-heavy pH shift that would be required if an alkaline electrolyte was used (figure 11). It is worth mentioning, however, that many electrodes are generally unstable in acidic media and quickly degrade above 1.30 *V*_RHE_ [81]. The authors annealed the MnO_x_ electrodes at 400°C for 2 h to improve chemical stability, although long-term electrode stability after electrolysis was not reported.

**Figure 12.**
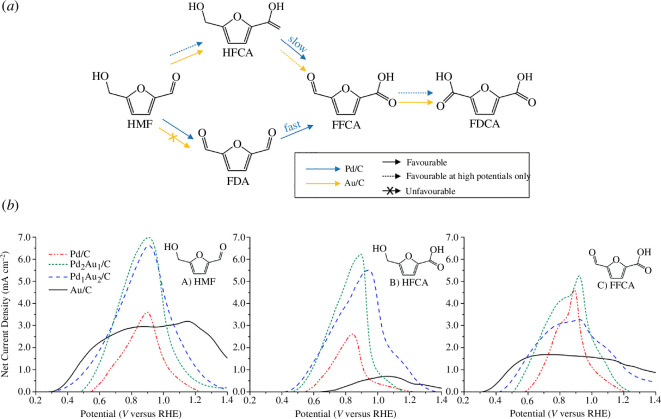
Electrochemical oxidation of HMF on Au_x_Pd_y_ electrodes. (*a*) Reported favoured reaction routes of HMF oxidation on Au/C and Pd/C anodes. (*b*) LSVs of various Pd and Au anodes using different starting substrates HMF, HMFCA and FFCA [82]. Reaction conditions: 0.1 M KOH (pH 13), room temperature, scan rate 50 mV s^−1^.

Other materials that have been reported for electrochemical HMF oxidation are bimetallic Pd_x_Au_y_ nanoparticles. A study by Chadderdon *et al*. [81] reported electrochemical FDCA production with Faradaic efficiencies as high as 83% in alkaline media using Pd_1_Au_2_ anodes at 0.90 *V*_RHE_. They suggested that Au and Pd nanoparticles on carbon are favourable for aldehyde and alcohol oxidation, respectively (figure 12), hence the complete oxidation on Au/C and Pd/C electrodes required a higher potential for oxidation activity (1.20 V_RHE_). When combined in a ratio of Pd_1_Au_2_, a synergistic effect can produce FDCA at lower potentials with high FDCA selectivity. A similar conclusion was reached by Kim *et al*. [83], whereby Au/C anodes were deemed to be selective for aldehyde oxidation and would form HMFCA as the main product. The authors proposed that this is the only step possible on Au/C cathodes; however, a work by Heidary & Kornienko [84] using operando Raman techniques concluded that DFF was the main product and not HMF. Au_3_Pd_2_ bimetallic species were also tested by Latsuzbaia *et al*. [77] with noticeable differences in electrochemical activity. Similarities remained with regard to high Faradaic efficiency (84%) and selectivity (92%) for FDCA production; however, a decrease in current density from 3.00 to 0.07 mA cm^−2^ was seen after 6 h at 1.15 *V*_RHE_, most likely due to Au poisoning, which was not reported by the previous study. The authors attribute this difference to the advantages of Au_x_Pd_y_ nanoparticles used by Chadderdon *et al*. as opposed to flat electrodes and wires, as well as using a higher pH of 13 as opposed to 12 in their study. The electrochemical activity was able to be regained after a pulse for 1 s at 2.0 *V*_RHE_, which likely delaminated the poisoning off the electrode.

**Scheme 2.**
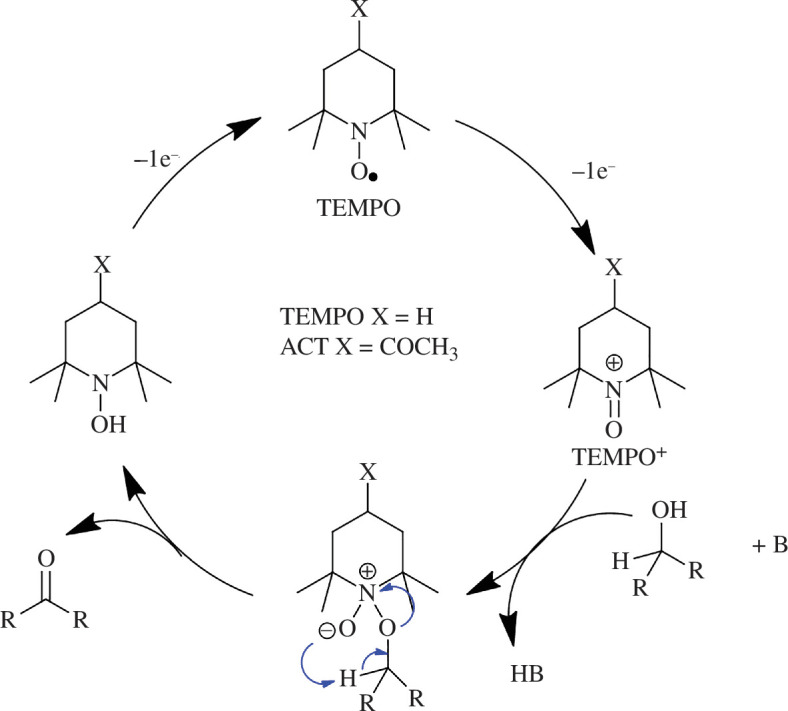
Electrochemical catalytic cycle of TEMPO demonstrating alcohol oxidation into aldehydes.

Lu *et al*. [85] reported Co-based spinel oxides (Co_3_O_4_, ZnCo_2_O_4_) displaying FDCA production with Faradaic efficiencies of over 90% and current densities reaching 16 mA cm^−2^, with currents as high as 60 mA cm^−2^ when the spinel oxides are placed on Ni foam supports. Interestingly, metal-free catalysts for electrochemical oxidation reactions can be prepared from ubiquitous elements such as carbon, oxygen, nitrogen and hydrogen. These include molecular catalysts such as the stable nitroxy radical 2,2,6,6-tetramethylpiperidine-1-oxyl (TEMPO). An applied potential oxidizes TEMPO into the oxoammonium cation (TEMPO^+^), which facilitates the addition of an alcohol for further reduction. The TEMPO radical can then be electrochemically restored to complete the oxidation cycle (scheme 2). Cardiel *et al*. [86] reported the conversion of HMF into FDCA using TEMPO in a homogenous borate buffer solution (pH 9.2) and produced FDCA with 96.7% Faradaic efficiency at 1.60 *V*_RHE_. Electron-withdrawing effects were explored using 4-acetyl-TEMPO (ACT), which resulted in FDCA being produced at a faster rate than TEMPO. They suggested that the electron-withdrawing acetyl group quickly forms the active oxoammonium species, which results in a more anodic potential required to form the active TEMPO^+^ species. The low price, high activity, low toxicity and high FDCA yields obtained using TEMPO make this catalyst appealing for a scale-up of FDCA production.

## 4. Side reactions of furanic compounds

**Scheme 3.**
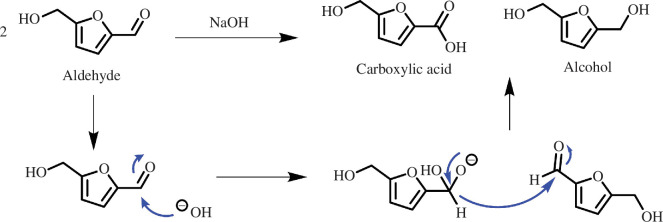
Mechanism for the base-catalysed Cannizzaro reaction starting from HMF.

As well as electrochemical degradation, non-electrochemical HMF degradation into humin products is also an issue. HMF is a relatively unstable aldehyde that rapidly degrades into humin products without any catalyst or applied potential. Van Zandvoort *et al*. [87] reported that humin products form through condensation reactions between the aldehyde group of one HMF molecule and the beta position of the furan ring of another. This phenomenon is problematic as these oligomers can poison the electrode surface preventing oxidation activity. It is important to mention that the aldehyde group in HMF is non-enolizable (absence of an alpha-H) and consequently makes HMF susceptible to the Cannizzaro reaction at high pH [88]. This is a disproportionation reaction that occurs when two aldehydes react with each other in an alkaline solution (scheme 3). Studies on the Cannizaro reaction of HMF in different alkaline solutions show that HMF is converted into FFCA and BHMF, as well as some humin products [5]. As an alkaline electrolyte is preferred for oxidation, the Cannizzaro reaction and humin formation are difficult to avoid completely but may be minimized by using electrodes that rapidly oxidize HMF into more stable products or finding a method that avoids using highly alkaline electrolytes.

## 5. Conclusion

In this review, we have provided specific examples of how electrification may potentially revolutionize the way clean fuels and chemicals are synthesized. The products obtained from upgrading furanic platform chemicals are viable for replacing chemicals sourced from petrochemical feedstock; however, several challenges need to be addressed first. Arguably, the biggest roadblock is the production rate of platform chemicals. While furfural is produced in quantities of about 0.25 million metric tonnes per year [89], the industrial scale-up of HMF production has not been yet established and so is not produced in the large quantities needed to completely replace petrochemical feedstocks. The work being done to produce industrial volumes of HMF is still in its relative infancy, although there have been ventures by the company Avantium [48] to produce large amounts of HMF and FDCA.

The electrosynthesis of useful chemicals is likely to become a frontier in lowering carbon footprints and directly utilizing green electricity. While some promising biomass electrovalorization processes have been reported here, most do not have high enough current densities required for a feasible industrial scale-up, have low selectivities and have not been reported in a continuous system that will likely be needed for the said industrial scale-up. In addition, the separation of products from the electrolyte will need to be addressed. We envisage that technoeconomic analyses coupled with life cycle assessment can assess the viability of these processes and guide scientists towards routes that make the largest possible impact [25].

## Data Availability

This article has no additional data.
